# Foot Morphology and Plantar Pressures in Elite Male Soccer Players—A Baropodometric On-Field Dynamic Assessment

**DOI:** 10.3390/sports13110408

**Published:** 2025-11-13

**Authors:** Pablo Vera-Ivars, Juan Vicente-Mampel, Oscar Fabregat-Andrés, Carlos Barrios

**Affiliations:** 1 School of Medicine and Health Sciences, Department of Podiatry, Catholic University of Valencia, 46001 Valencia, Spain; fp.vera@ucv.es; 2 School of Medicine and Health Sciences, Department of Physiotherapy, Catholic University of Valencia, 46009 Valencia, Spain; juan.vicente@ucv.es; 3 School of Medicine and Health Sciences, Department of Medicine, Catholic University of Valencia, 46001 Valencia, Spain; oscar.fabregat@ucv.es; 4 Institute for Research on Musculoskeletal Disorders, School of Medicine and Health Sciences, Catholic University of Valencia, 46001 Valencia, Spain

**Keywords:** baropodometry, soccer, foot plantar pressures, BioFoot^®^

## Abstract

Introduction: Numerous overuse injuries affecting the lower limbs of elite athletes have been associated with biomechanical alterations in plantar loading of the foot. This study aimed to analyze the plantar pressure distribution in elite male soccer players and its relationship with various morphological and functional factors, including foot type, metatarsal and digital alignment, and on-field position. Material and Method: Dynamic foot pressure measurements were obtained from 21 soccer players who participated in the UEFA Champion League. The participants had an average age of 27 years, with an average height of 180.9 cm, weight of 76.9 kg, and BMI of 23.4. An insole system (BioFoot/IBV) with telemetry transmission was employed to record plantar loading patterns during normal gait and running. Results: During the support or contact phase, the central and medial metatarsal areas exhibited the highest peak pressure under both walking and running conditions. When walking, the right foot exerted 13–60% more pressure on the outer metatarsal and toe areas. The left foot experienced up to 13% more peak pressure in the middle metatarsal area. During running, the total pressure difference between the feet ranged from −8% to +19%. The right foot usually had more peak pressure on the heel and first toe. In players with valgus feet, the pressure in the central metatarsal area increased from 1086 kPa (walking) to 1490 kPa (running), representing a 37% increase. Conversely, in players with cavus-varus feet, the pressure in this central area increased from 877 kPa to 1804 kPa, a 105% increase. Conclusions: Foot morphology and playing position significantly influenced the plantar pressure patterns in elite soccer players. The central metatarsal region bears the highest load, particularly during running, with distinct variations across foot types and field positions. These findings highlight the need for individualized biomechanical assessments to prevent overuse injuries and optimize performance.

## 1. Introduction

Soccer exposes players to repeated high magnitude impacts and rapid changes in direction that concentrate mechanical loads on specific plantar regions of the foot [1]. These sport-specific demands have been associated with overuse pathology across the lower limb, wherein deviations in plantar loading may exacerbate symptoms by altering the transmission of stresses through articular structures and periarticular soft tissues [2,3,4]. Although plantar pressure analysis is routinely employed to characterize foot loading and inform therapeutic or performance interventions, evidence focused specifically on professional soccer athletes—particularly analyses stratified by tactical role and intrinsic foot morphology—remains comparatively scarce [5,6]. Elite players differ biomechanically from amateurs, exhibiting higher peak ground reaction forces, more frequent and intense accelerations/decelerations, and distinct movement patterns that lead to specific musculoskeletal adaptations [7]. Consequently, studies conducted on amateur populations may not fully capture the loading magnitudes and positional demands present at the elite level [8]. This distinction underscores the need to investigate plantar pressure patterns, specifically in professional soccer players, highlighting a clear research gap that our study aimed to address.

Players’ biomechanical demands vary with their tactical role and on-field position [9,10]. Central defenders typically cover the least total and high-speed distance and execute the fewest accelerations/decelerations; their activity is dominated by brief, reactive accelerations from near-static positions and frequent high-magnitude decelerations during marking and duels, with limited maximal sprinting. Midfielders (central and wide) accumulate the greatest total distance and high intensity running, characterized by numerous moderate accelerations/decelerations throughout the match but fewer maximal sprints than wingers or forwards. Forwards cover less total distance yet produce more short, high-intensity bursts and attain higher peak speeds, with loads marked by repeated accelerations from rolling starts and sharp decelerations after attacking actions. These positional profiles are consistent across leagues and are modulated by team tactical systems. Because position-specific biomechanics produce distinct foot–ground interactions, plantar-pressure mapping may reveal where the load concentrates and how it is spread. Measuring these distributions links external workload to tissue-level stress, enabling targeted injury prevention, footwear/orthotic tuning, and role-specific training/return-to-play decisions.

Baropodometric assessment provides objective measurements of plantar load under static and dynamic conditions and is widely applied in both clinical and sports contexts [11,12,13,14]. In-shoe pressure systems enable the collection of multiple consecutive steps during natural movement, producing gait and running data that represent real locomotion more accurately than single-step platforms [11,12,15]. This feature is particularly useful for soccer, where rapid and high-intensity movements make accurate peak load detection essential for performance and injury prevention [12,13]. Understanding plantar pressure behavior through these technologies helps interpret how loading patterns relate to performance and injury risk in elite soccer. Within athletic environments, in-shoe systems are widely implemented to quantify plantar loading during running and sport-specific maneuvers [16,17], evaluate the influence of footwear and playing surface [1,18], and monitor symmetry, center-of-pressure progression, and fatigue-related shifts—parameters that support both performance enhancement and injury mitigation strategies [3,16]. However, despite their growing use in other sports, studies integrating on-field in-boot pressure data with player-specific factors such as foot morphology and tactical position remain limited in elite soccer [19].

Existing soccer research has frequently emphasized discrete skills (e.g., cutting, accelerating, jumping, kicking, sprinting) or relied on small, heterogeneous, or non-professional samples, thereby limiting the generalizability of findings to elite competition contexts [3,17,18]. Moreover, most investigations on technologies for plantar pressure measurement during soccer tasks have focused on the effects of footwear, cleat type, surface characteristics, and isolated athletic activities [1,17,18,20]. Few studies have concurrently examined how intrinsic morphology (foot type and forefoot formulas) and extrinsic role demands (defender, midfielder, forward) shape plantar loading patterns during soccer-specific locomotor tasks [5,6]. Digital alignment modulates plantar pressure distribution, with toe malalignment increasing focal loading under metatarsal heads [21]. Addressing these gaps could sharpen risk profiling (e.g., forefoot hyperpressures and metatarsal stress risk) and guide individualized interventions (orthotic tuning, cleat configuration, and workload management) tailored to tactical roles and foot structures [17,18]. Accordingly, the primary aim of this study was to characterize the dynamic plantar pressure distribution in elite male professional soccer players from a top-tier Spanish club competing in the UEFA Champions League using an in-shoe pressure system during both walking and running. Additionally, baropodometric patterns associated with morphological (foot type, metatarsal, and digital alignment) and functional (player position) factors were analyzed. Our exploratory hypothesis was that (i) foot type and forefoot regions—particularly the central and medial metatarsals—would modulate regional loading patterns when walking and running; and (ii) playing position would be independently associated with distinct pressure profiles reflecting role-specific movement demands.

## 2. Materials and Method

### 2.1. Study Design

A retrospective, descriptive, and comparative cross-sectional study was conducted involving a cohort of male soccer players with substantial experience in elite European leagues. Data collection was retrospectively analyzed, and potential selection and measurement biases were minimized by including all eligible players assessed during the same testing period under standardized conditions. The baropodometric studies were performed in line with the ethical standards of the Declaration of Helsinki [22]. The study’s design and participant progression followed the STROBE guidelines [23].

### 2.2. Participants and Settings

A cohort of 21 professional active first-level football players was assessed in this study (Table 1). All soccer players who were enrolled in the team during the period of study underwent a foot clinical assessment as part of routine medical evaluations performed by the same specialist in sports traumatology. Participants with a medical history of previous surgeries on the lower limb or severe fractures within the six months preceding evaluation were excluded. Data collection was conducted from March 2016 to May 2025 at the Artes Timefit Clinic. The plantar pressure evaluation was performed by the same podiatrist with expertise in biomechanical assessments, possessing over a decade of professional experience. Plantar pressures were therefore recorded in a standardized manner always following the same protocol. Pressure recording was performed during training sessions and not at the time of competition. Out of the 30 players who initially underwent the plantar pressure recording (12 defenders and goalkeepers, 12 midfielders, and 6 forwards), a group of 9 were excluded for different medical reasons that could affect plantar load, leaving only 21 players to be finally included in the study (Table 2).

### 2.3. Variables and Data Sources

The physical examination protocol comprised a biomechanical assessment conducted prior to the procedure for recording plantar pressures. The clinical profile encompasses the following parameters: foot type (cavus-varus, valgus), metatarsal alignment (index plus, index plus-minus), toe alignment (Greek, squared), leg length inequality (present or absent), and player position (midfielders, defenders, forwards). The distribution of these parameters among players is presented in Table 2.

### 2.4. Quantitative Variables (BIOFOOT^®^)

**Figure 1:**
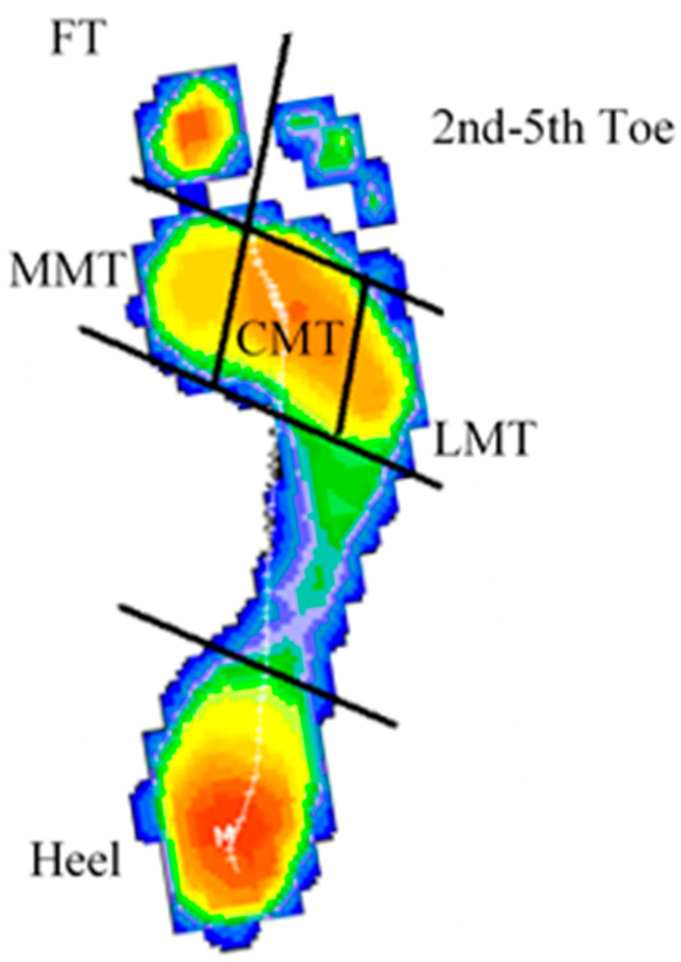
Different anatomic plantar areas where the pressures were recorded. FT, first toe; MMT, medial metatarsal head; CMT, second and third metatarsal heads; LMT, lateral or fifth metatarsal head. Red colors indicated higher pressures and blue lower pressures.

The BioFoot system from the Valencia Institute of Biomechanics (IBV, Valencia, Spain) was used to collect the foot pressure data [24]. FIFA approved this system for checking field quality (www.fifa.com, accessed on 8 August 2025). The BioFoot/IBV in-shoe system has been demonstrated to be a reliable and reproducible instrument for evaluating plantar pressure distribution under dynamic conditions [25]. Nevertheless, it is recognized that, to date, the system has not been directly compared with gold-standard baropodometric platforms such as Pedar-X, Novel, or Tekscan [26,27]. It includes two insoles with 64 sensors each, recorded at 50–250 Hz [28]. The frequency used in all of the baropodometric studies in this sample of soccer players was 150 Hz, and the estimated impedance was 256 kΩ. Pressures were measured in kilopascals [16]. Measurements were performed in a well-maintained soccer field [5]. The mean peak pressures obtained from 10 s assessment were considered for analysis. Players wore their usual soccer boots. In 11-player soccer, boots have 10–11 cleats. The insoles were placed between the foot and shoe. Two measurements were performed: (i) while walking normally and (ii) while running speed was self-selected by the player, avoiding sprinting, and was not monitored. The insole sensors were divided into eight areas matching the foot parts (Figure 1), a method used by other researchers [29,30]. This study focused specifically on plantar peak pressure distribution, rather than the full spectrum of ground reaction forces (e.g., vertical GRF, torsional moments, or shear forces). The pressure data were analyzed using factors such as player height, weight, footwear, foot type, metatarsal and digital formula, dominant limb, and field position (forward, midfielder, defenders).

### 2.5. Bias

To mitigate potential biases inherent in retrospective study designs, such as selection and information biases, stringent inclusion and exclusion criteria were implemented, and data quality was rigorously assessed for completeness and consistency. Furthermore, confounding variables, including training load [31] and injury history, were accounted for during data analysis to enhance the reliability of the findings. Moreover, player speed was not adjusted in any instance, thereby facilitating a more physiological gait dynamic. An adaptation phase of 25–50 steps was provided to each player prior to data collection with the insoles.

### 2.6. Statistical Methods

Continuous variables are presented as the mean ± standard deviation (SD) and 95% confidence intervals (CIs). CIs were interpreted as proposed by O’Brien and Long Yi [32]. The assumption of normality was assessed using the Kolmogorov–Smirnov test. Nonparametric variables were compared using the Mann–Whitney U test or Kruskal–Wallis test (for comparisons of more than two groups). Normally distributed variables were compared using the Student’s *t*-test or one-way ANOVA, as appropriate. Post hoc analyses were performed using Bonferroni correction. For the Student’s *t*-test, small, medium, and large effect size diagnostic thresholds were considered of 0.2, 0.5, and 1.0, respectively [33]. The strength of the relationship between the variables was examined using the Pearson correlation coefficient and/or Spearman correlation coefficient (for non-compliance with the normality assumption). Effect size intervals for r were provided according to the following guidelines: small, medium, and large effects were considered 0.3, 0.5, and 0.6, respectively [33]. Additionally, paired comparisons between walking and running were analyzed using paired *t*-tests or Wilcoxon tests, and the percentage changes were calculated for each plantar region. All analyses were conducted using SPSS version 28 (IBM Corp., Armonk, NY, USA) and JASP (Amsterdam, The Netherlands, Version 0.15.0.0.0; JASP Team, 2023). Statistical analysis was performed by a researcher not involved in data collection using coded data to ensure blinding. Statistical significance was set at *p* < 0.05.

## 3. Results

**Figure 2:**
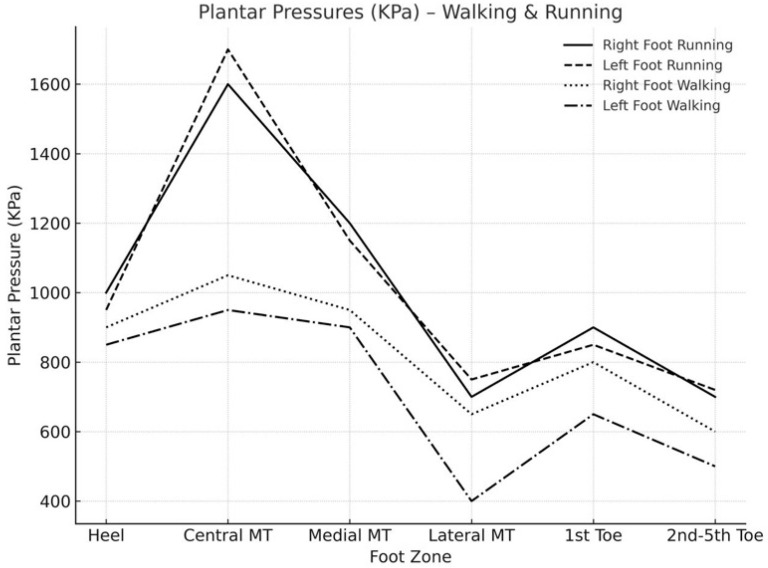
Plantar peak pressures in the anatomic areas recorded in both walking and running conditions. Right and left foot are presented independently.

During ambulation, both walking and running, the greatest peak pressures were recorded at the central metatarsal heads, followed by the first metatarsal and the heel. Differences between the left and right feet were assessed using paired statistical tests, as reported in Table 3. No statistically significant differences were observed in any of the plantar regions during walking or running. The values presented denote the average peak plantar pressures recorded per step, aggregated across all measured anatomical regions, as opposed to representing cumulative or continuous loads over multiple steps. Table 3 provides a summary of the pressures recorded in the six anatomical areas corresponding to the right and left feet under both walking and running conditions. The same pattern of plantar pressure distribution was evident during both walking and running (Figure 2). When walking, the right foot exerted 13–60% more pressure on the outer metatarsal and toe areas. The left foot experienced up to 13% more pressure in the middle metatarsal area. During running, the pressure difference between the feet ranged from −8% to +19%. The right foot usually had more pressure on the heel and first toe

In both conditions, the highest pressure was observed in the region corresponding to the central metatarsal heads, followed by the first metatarsal loading area, heel, first toe, and finally, the lateral metatarsal bones. As anticipated, there was an increase in pressure across all plantar regions during running compared to walking. This increase was particularly pronounced in the central area, with an average increment of 566.2 kPa for the right foot and 561.9 kPa for the left foot. Total plantar pressures showed no direct correlation with player weight. When analyzed by anatomical region, only the heel and lateral metatarsal areas exhibited a positive correlation with weight, with stronger correlations observed in the right foot (Table 4).

**Figure 3:**
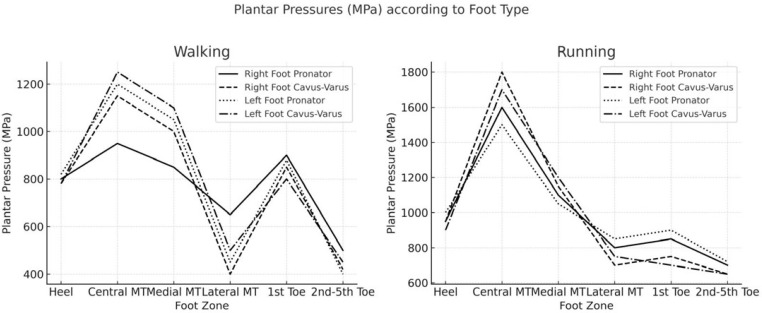
Plantar peak pressures during walking and running depending on the foot type.

In the examination of foot morphology among this cohort of soccer players, two distinct types were identified: pronator-valgus and cavus-varus feet. No significant differences were observed in the mean total plantar pressure loading between these foot types (*p* > 0.05). However, the pronator-valgus foot consistently exhibited the highest total pressures during both walking and running. Notably, foot type influenced the plantar pressure pattern, specifically the distribution and variation of plantar pressures across different analyzed areas. Among players with a pronator-valgus foot type, the central metatarsal area experienced the most substantial increase in plantar pressure during running, with measurements of 404.5 kPa in the right foot (a 37% increase) and 385.8 kPa in the left foot (a 34% increase). Conversely, in players with a cavus-varus foot type, the central metatarsal area of the right foot exhibited a pressure increase from 877.8 to 1763.6 kPa, representing a 101% increase (Figure 3). Additionally, the pressure at the right first toe decreased by 24% during running in individuals with a cavus-varus foot type.

Regarding metatarsal alignment, soccer players with index plus-minus alignment exhibited higher total plantar pressure at both walking and running speeds, with averages of 4090 ± 1497.9 kPa and 5543.1 ± 1708.2 kPa, respectively, whereas the index minus feet showed lower pressures (3850 ± 1420 kPa and 4804.2 ± 1676.3 kPa, respectively). The main difference occurred in the medial metatarsal region, where pressure decreased during running in the index plus minus feet. This may be due to first-ray geometry: a relatively longer first metatarsal in the index plus-minus feet allows medial-to-central load transfer during push-off, while a shorter first metatarsal in the index minus feet shifts forces laterally, concentrating stress on the central metatarsals. Such biomechanical considerations are likely to explain the regional pressure variations observed under high-speed conditions. However, feet with index minus displayed a different behavior, with an increase in loading while running. The plantar pressure distribution was also influenced by the digital formula. Greek feet experienced higher total plantar pressure than square feet (4241.4 ± 1444.4 versus 3504 ± 1449.1 kPa), although these differences were not statistically significant (*p* > 0.05). Nevertheless, there was a 12% decrease in loading at the first toe in square feet while running, and an 18% increase in Greek feet. Greek feet also experienced higher pressure during running. In the central metatarsal area, Greek feet showed a 43% increase in pressure between walking and running, whereas the increase was 57% in square feet.

**Figure 4:**
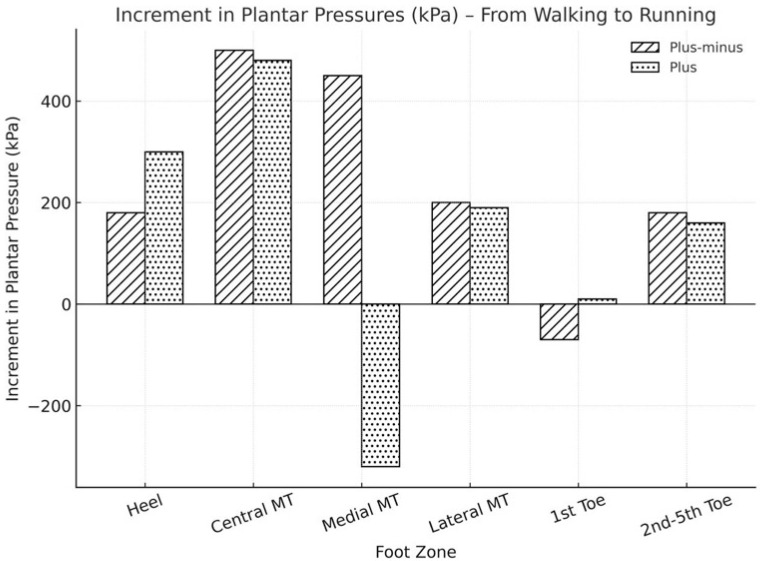
Increment in plantar peak pressures from walking to running depending on the metatarsal alignment.

**Figure 5:**
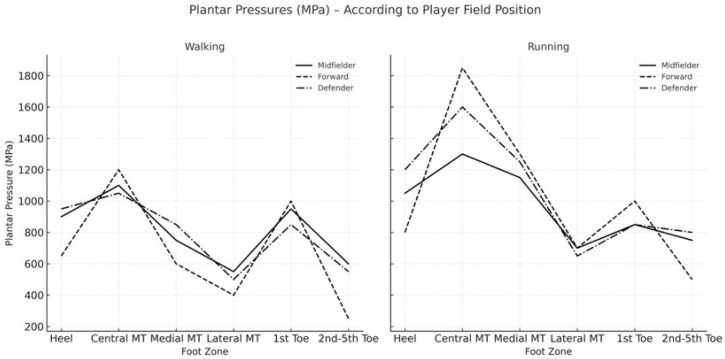
Peak plantar pressures during walking and running depending on the different players’ position into the soccer field (right foot).

**Figure 6:**
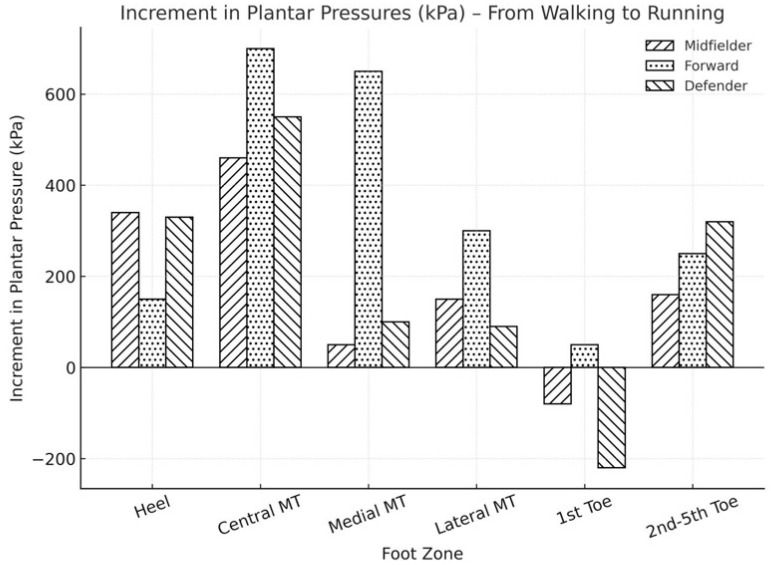
Increment in peak plantar pressures from walking to running in the right foot according to the different players’ position into the soccer field.

The analysis of data pertaining to plantar pressure distribution relative to line-up position on the field yielded noteworthy findings (Figure 4). Under walking conditions, the most significant differences were observed between midfielders and forwards. Forwards exhibited increased plantar pressures in the central metatarsal region and reduced pressure in the medial and lateral metatarsal areas, as well as at the small toes. In contrast, midfielders demonstrated the highest pressure in the medial metatarsal area. During running, forwards showed the greatest increase in pressure in both the central and medial metatarsal regions (Figure 5). Defenders experienced higher increments in heel pressure and a reduction in pressure at the first toe (Figure 6).

## 4. Discussion

The present study, conducted in professional soccer players using an in-shoe system under real-world testing environments, confirmed a substantial rise in plantar pressure from walking to running, with a predominant concentration over the central metatarsal region and secondary increases at the medial forefoot. Despite this forefoot emphasis, the bilateral differences were generally small, indicating an overall symmetrical loading pattern between the feet, particularly during running. The largest asymmetries appeared at the first toe and lateral metatarsal areas during walking, suggesting subtle dominance-related adaptations under slower controlled movements rather than structural imbalance. Interestingly, in contrast with the previous literature [34,35], body mass did not explain the total plantar load, although it was modestly related to region-specific pressures (heel and lateral metatarsals), reinforcing that morphology and task demands dictate regional loading more than global anthropometrics [6]. While our analysis did not identify a significant correlation between total plantar pressure and body weight, regional analyses revealed notable associations, particularly in the heel and lateral metatarsal regions. This pattern indicates a compensatory redistribution of load across various plantar areas, reflecting the intricate interplay between foot morphology, running mechanics, and localized loading. Recognizing this distinction is crucial to avoid interpreting the absence of a total pressure correlation as biomechanically irrelevant, underscoring the importance of region-specific assessments in elite soccer players. Beyond its descriptive value, this pattern underscores the functional role of the central rays as the primary propulsive “pivot” during fast locomotion in soccer, a role likely magnified by cleated footwear and natural grass interaction [20,36]. The use of the BioFoot/IBV device inside the players’ usual boots, capturing multiple natural steps without platform targeting, strengthens the external validity of these observations for high-level competitions.

In line with previous research, pronator-valgus feet exhibited the highest total plantar loads across gait conditions, whereas cavus-varus feet demonstrated the most pronounced redistribution with increased speed, including nearly 100% increases at the central metatarsals and relative reductions at the hallux during running [4,5]. These results corroborate earlier studies reporting that forefoot morphology strongly influences regional loading patterns, although the magnitude of change observed in elite soccer players is greater than that in less trained populations. Forefoot geometry further modulated stress distribution: players with an index plus-minus alignment showed higher total pressures, particularly in the medial forefoot, whereas the digital (toe) formula determined whether propulsive forces were preferentially borne by the hallux or shifted toward the central rays [37]. Mechanistically, these findings suggest that metatarsals II–III accept a disproportionate share of propulsive forces when the first-ray contribution is limited by either morphology or high-speed demands, concentrating stress in the anterocapital region [17,18]. Measured pressures exceeded 3000 kPa during running, substantially higher than the ~700 kPa typically reported in non-athletic populations also during running, highlighting the marked biomechanical adaptations of elite soccer players [37]. Practically, these data indicate that the central and medial metatarsals, and depending on toe morphology, the hallux, should be prioritized in monitoring strategies during high-load phases of training and competition, and may inform the design of targeted orthotics, footwear adjustments, and conditioning programs to mitigate overuse injury risk [6].

Plantar pressure patterns vary according to the players’ on-field roles, reflecting position-specific movement demands [6]. Forwards showed higher central and medial forefoot pressures, consistent with acceleration and finishing actions. Midfielders exhibited increased medial forefoot loading aligned with sustained multidirectional shuttling. Defenders displayed greater rearfoot pressure, which was likely related to braking and clearance movements. The results should not be over interpreted due to the small sample size per subgroup. However, when interpreting variations in plantar pressure, it is essential to exercise caution, as uncontrolled variables such as running speed, task type, and individual running style may have influenced the observed loading patterns.

### Strengths and Limitations

Methodologically, functional on-field evaluation of elite athletes with in-boot instrumentation is a major strength of this study, increasing the external validity relative to single-step platform testing and capturing step-to-step variability relevant to high-intensity soccer tasks [11,12]. At the same time, several limitations should be considered to avoid unappropriated generalization: a single top-tier squad, heterogeneous boot models and stud layouts, and the absence of strict speed control (which also preserves real-world behavior) may introduce variance; and the present tasks were linear (walking/running) rather than football-specific maneuvers (cuts, sprints, striking) that often amplify medial forefoot loading [17]. Extending this approach to sport-specific actions, incorporating standardized or instrumented speed metrics when appropriate, and prospectively testing orthotic or footwear modifications could clarify causal pathways from morphology and role demands to hyperpressure-related symptoms. A potential limitation of this study is the sample size, which, although representative of elite athletes, may have limited the statistical power to detect small but meaningful differences between plantar regions or between foot types. The post hoc power analysis revealed moderate-to-low statistical power (ranging from approximately 0.20 to 0.45 across regions), suggesting that some nonsignificant results might reflect type II error rather than the absence of true biomechanical differences. Future studies with larger cohorts or repeated-measures designs are warranted to confirm these trends and strengthen the generalizability of the present findings.

## 5. Conclusions

Overall, this study highlights the critical role of foot morphology in determining plantar pressure patterns among elite male soccer players. The metatarsal region, particularly the central area, endures the greatest mechanical stress during dynamic activities, especially running. Players with cavus-varus and valgus feet exhibited markedly different loading responses, emphasizing the need for individualized biomechanical assessment and preventive strategies. Importantly, these findings underscore the potential for the longitudinal monitoring of plantar pressures in elite athletes, enabling the early detection of abnormal load patterns, informed adjustments to training and footwear, and targeted interventions by orthotic new designs and changes in cleat configurations. All of these possible interventions could optimize performance while reducing the risk of overuse injuries.

## Figures and Tables

**Table 1:** Demographic profile of the soccer players included in the study.

Variables	Mean	±SD	Minimum	Maximum
Age	27.1	±4.1	20.0	33.0
BMI	23.4	±1.3	21.0	26.0
Height (cm)	180.9	±4.2	173.0	187.0
Weight (kg)	76.9	±3.9	70.0	84.0
Foot size (inches)	27.2	±0.9	25.1	29.2

**Table 2:** Biological and clinical profile of the soccer players included in the study.

Clinical Parameter	Type	Frequency
Foot type	Pronator-Valgus	12 (57.1%)
	Cavus-Varus	9 (42.9%)
Toe alignment	Greek	16 (76.2%)
	Squared	5 (23.8%)
Metatarsal alignment	Index Plus	15 (71.4%)
	Index Plus minus	6 (28.6%)
Dexterous/left-handed	Dexterous	19 (90.5%)
	Left handed	2 (9.5%)
Leg length inequality	Present	1 (4.8%)
	Absent	20 (95.2%)
	Defenders	7 (33.3%)
Player position on the field	Midfielders	8 (38.1%)
	Forwards	6 (28.6%)

**Table 3:** Comparative analysis of plantar pressures (Mean ± SD, 95% CI, *p*-values, and Cohen’s d) between the left and right feet under walking and running conditions.

	Left Foot		Right Foot		
Anatomical Region	Mean ± SD	95% CI	Mean ± SD	95% CI	*p*-Value; Cohen’s d
Walking					
Heel	735.2 ± 252.7	[620.3, 850.1]	729.4 ± 293.0	[596.7, 862.1]	0.868; 0.02
Central Metatarsal	1123.8 ± 581.3	[859.3, 1388.3]	978.6 ± 374.0	[804.5, 1152.7]	0.442; 0.30
Medial Metatarsal	852.8 ± 767.5	[510.7, 1194.9]	857.1 ± 790.9	[492.2, 1222.0]	0.926; −0.01
Lateral Metatarsal	376.2 ± 195.3	[288.5, 463.5]	491.4 ± 274.6	[362.5, 620.3]	0.084; −0.48
First Toe	560.9 ± 354.6	[398.0, 723.0]	900.0 ± 687.2	[585.1, 1214.9]	0.083; −0.62
2nd–5th Toe	325.0 ± 228.0	[223.8, 426.2]	399.4 ± 357.0	[237.4, 561.4]	0.163; −0.25
Running					
Heel	871.4 ± 511.8	[638.3, 1104.5]	988.9 ± 516.2	[751.2, 1226.6]	0.190; −0.23
Central Metatarsal	1685.7 ± 565.1	[1431.3, 1940.1]	1544.8 ± 522.6	[1306.9, 1782.7]	0.656; 0.26
Medial Metatarsal	1035.7 ± 515.8	[807.6, 1263.8]	1123.8 ± 674.7	[819.3, 1428.3]	0.840; −0.14
Lateral Metatarsal	740.0 ± 455.2	[529.0, 951.0]	683.8 ± 399.8	[507.3, 860.3]	0.768; 0.13
First Toe	697.1 ± 360.7	[534.0, 860.2]	828.6 ± 521.9	[592.3, 1065.0]	0.211; −0.29
2nd–5th Toe	691.9 ± 529.4	[450.5, 933.3]	632.5 ± 459.1	[420.1, 844.9]	0.378; 0.12

Note. Values are presented as the mean ± standard deviation (SD) with 95% confidence interval (CI). *p*-values correspond to comparisons between the left and right feet in each anatomical region. Cohen’s d represents the standardized effect size (small: 0.2, moderate: 0.5, large: 1.0).

**Table 4:** Correlations between the player’s weight and the peak plantar pressures on both feet and during walking and running conditions.

Anatomical Region	Statistical Analysis	Walking		Running	
		Right Foot	Left Foot	Right Foot	Left Foot
Heel	Pearson correlation	0.49 * (IC95% [0.06, 0.77]; r^2^ = 0.24)	0.44 * (IC95% [0.00, 0.74]; r^2^ = 0.19)	0.56 ** (IC95% [0.16, 0.80]; r^2^ = 0.31)	-
	Sig. (bilateral)	0.02	0.04	0.01	-
Lateral Metatarsal	Pearson correlation	0.49 * (IC95% [0.06, 0.77]; r^2^ = 0.24)	-	0.51 * (IC95% [0.09, 0.77]; r^2^ = 0.26)	-
	Sig. (bilateral)	0.02	-	0.01	-

Note. ** Significant correlation at the 0.05 level; * Significant correlation at the 0.01 level. Confidence intervals (95%) were computed using Fisher’s z transformation. Effect sizes (r^2^) represent the proportion of variance explained. Correlation coefficients were interpreted as small (r = 0.3), medium (r = 0.5), and large (r ≥ 0.6) effects.

## Data Availability

The original contributions presented in the study are included in the article, further inquiries can be directed to the corresponding author.

## Abbreviations

The following abbreviations are used in this manuscript:

IBV	Instituto de Biomecánica de Valencia
BMI	Body Mass Index
FT	First Toe
MMT	Medial Metatarsal
CMT	Central Metatarsals
CI	Confidence intervals
LMT	Lateral Metatarsal
SPSS	Statistical Package for the Social Sciences
JASP	Jeffreys’s Amazing Statistics Program
STROBE	Strengthening the Reporting of Observational Studies in Epidemiology
FIFA	Fédération Internationale de Football Association
kPa	Kilopascal
UEFA	Union of European Football Associations
SD	Standard Deviation (Desviación estándar)

## References

[1] Queen R.M., Haynes B.B., Hardaker W.M., Garrett W.E. (2007). Forefoot loading during three athletic tasks. Am. J. Sports Med..

[2] Oztekin H.H., Boya H., Ozcan O., Zeren B., Pinar P. (2009). Foot and ankle injuries and time lost from play in professional soccer players. Foot.

[3] Mandorino M., Figueiredo A.J., Gjaka M., Tessitore A. (2023). Injury incidence and risk factors in youth soccer players: A systematic literature review. Part I: Epidemiological analysis. Biol. Sport.

[4] Fernández-Seguín L.M., Díaz Mancha J.A., Sánchez Rodríguez R., Escamilla Martínez E., Gómez Martín B., Ramos Ortega J. (2014). Comparison of plantar pressures and contact area between normal and cavus foot. Gait Posture.

[5] Lozano-Berges G., Matute-Llorente Á., Gómez-Bruton A., Alfaro-Santafé V., González-Agüero A., Vicente-Rodríguez G., Casajús J.A. (2019). Plantar pressures in male adolescent soccer players and their associations with bone geometry and strength. J. Sports Med. Phys. Fit..

[6] Hawrylak A., Brzeźna A., Chromik K. (2021). Distribution of plantar pressure in soccer players. Int. J. Environ. Res. Public Health.

[7] Burns G.T., Kozloff K.M., Zernicke R.F. (2020). Biomechanics of Elite Performers: Economy and Efficiency of Movement. Kinesiol. Rev..

[8] Pillitteri G., Giustino V., Petrucci M., Rossi A., Bellafiore M., Thomas E., Iovane A., Bianco A., Palma A., Battaglia G. (2023). External load profile during different sport-specific activities in semi-professional soccer players. BMC Sports Sci. Med. Rehabil..

[9] Sarmento H., Martinho D.V., Gouveia É.R., Afonso J., Chmura P., Field A., Savedra N.O., Oliveira R., Praça G., Silva R. (2024). The influence of playing position on physical, physiological, and technical demands in adult male soccer matches: A systematic scoping review with evidence gap map. Sports Med..

[10] González-Rodenas J., Moreno-Pérez V., Castaño-Zambudio A., López-Del Campo R., Nevado F., Coso J.D. (2025). Locomotor characteristics of intense accelerations according to playing position in top Spanish football teams during competition. Biol. Sport.

[11] Burnie L., Chockalingam N., Holder A., Claypole T., Kilduff L., Bezodis N. (2024). Testing protocols and measurement techniques when using pressure sensors for sport and health applications: A comparative review. Foot.

[12] Ramirez-Bautista J.A., Huerta-Ruelas J.A., Chaparro-Cardenas S.L., Hernandez-Zavala A. (2017). A review in detection and monitoring gait disorders using in-shoe plantar measurement systems. IEEE Rev. Biomed. Eng..

[13] Baumfeld D., Baumfeld T., da Rocha R.L., Macedo B., Raduan F., Zambelli R., Silva T.A.A., Nery C. (2017). Reliability of baropodometry in the evaluation of plantar load distribution: A transversal study. Biomed. Res. Int..

[14] Zulkifli S.S., Loh W.P. (2020). A state-of-the-art review of foot pressure. Foot Ankle Surg..

[15] Drăgulinescu A., Drăgulinescu A.-M., Zincă G., Bucur D., Feieș V., Neagu D.-M. (2020). Smart Socks and In-Shoe Systems: State-of-the-Art for Two Popular Technologies for Foot Motion Analysis, Sports, and Medical Applications. Sensors.

[16] Valldecabres R., Richards J., De Benito A.M. (2022). The effect of match fatigue in elite badminton players using plantar pressure measurements and the implications to injury mechanisms. Sports Biomech..

[17] Wong P., Chamari K., Mao D.W., Wisløff U., Hong Y. (2007). Higher plantar pressure on the medial side in four soccer-related movements. Br. J. Sports Med..

[18] Eils E., Streyl M., Linnenbecker S., Thorwesten L., Völker K., Rosenbaum D. (2004). Characteristic plantar pressure distribution patterns during soccer-specific movements. Am. J. Sports Med..

[19] Hotfiel T., Golditz T., Wegner J., Pauser J., Brem M., Swoboda B., Carl H.-D. (2020). A cross-sectional study on foot loading patterns in elite soccer players of different ages. J. Back Musculoskelet. Rehabil..

[20] Silva D.C.F., Santos R., Vilas-Boas J.P., Macedo R., Montes A.M., Sousa A.S.P. (2017). Influence of cleat–surface interaction on performance and injury risk in soccer: A systematic review. Appl. Bionics Biomech..

[21] Chow T.H., Chen Y.S., Hsu C.C. (2021). Relationships between plantar pressure distribution and rearfoot alignment in Taiwanese college athletes with plantar fasciopathy during static standing and walking. Int. J. Environ. Res. Public Health.

[22] Duncan N. (2013). Declaration of Helsinki. World Med. J..

[23] STROBE Checklists [Internet]. https://www.strobe-statement.org/checklists/.

[24] Martínez Assucena A., Sánchez Ruiz M.D., Barrés Carsí M., Pérez Lahuerta C., Guerrero Alonso A., Soler Gracia C. (2003). Un nuevo método de evaluación diagnóstica y terapéutica de las patologías del pie basado en las plantillas instrumentadas Biofoot/IBV. Rehabilitación.

[25] Martínez-Nova A., Cuevas-García J.C., Pascual-Huerta J., Sánchez-Rodríguez R. (2007). BioFoot^®^ in-shoe system: Normal values and assessment of reliability and repeatability. Foot.

[26] Withers R.V., Perrin B.M., Landorf K.B., Raspovic A. (2023). Offloading effects of a removable cast walker with and without modification for diabetes-related foot ulceration: A plantar pressure study. J. Foot Ankle Res..

[27] Blades S., Jensen M., Stellingwerff T., Hundza S., Klimstra M. (2023). Characterization of the Kinetyx SI wireless pressure-measuring insole during benchtop testing and running gait. Sensors.

[28] Escamilla-Martínez E., Gómez-Martín B., Fernández-Seguín L.M., Martínez-Nova A., Pedrera-Zamorano J.D., Sánchez-Rodríguez R. (2020). Longitudinal analysis of plantar pressures with wear of a running shoe. Int. J. Environ. Res. Public Health.

[29] Bisiaux M., Moretto P. (2008). The effects of fatigue on plantar pressure distribution in walking. Gait Posture.

[30] Wiegerinck J.I., Boyd J., Yoder J.C., Abbey A.N., Nunley J.A., Queen R.M. (2009). Differences in plantar loading between training shoes and racing flats at a self-selected running speed. Gait Posture.

[31] Wang C., Kaufman J.S., Steele R.J., Shrier I. (2024). Target trial framework for determining the effect of changes in training load on injury risk using observational data: A methodological commentary. BMJ Open Sport Exerc. Med..

[32] O’Brien S.F., Yi Q.L. (2016). How do I interpret a confidence interval?. Transfusion.

[33] Zieliński G. (2025). Effect Size Guidelines for Individual and Group Differences in Physiotherapy. Arch. Phys. Med. Rehabil..

[34] Tománková K., Přidalová M., Svoboda Z., Cuberek R. (2017). Evaluation of plantar pressure distribution in relationship to body mass index in Czech women during walking. J. Am. Podiatr. Med. Assoc..

[35] Ohlendorf D., Kerth K., Osiander W., Holzgreve F., Fraeulin L., Ackermann H., Groneberg D.A. (2020). Standard reference values of weight and maximum pressure distribution in healthy adults aged 18–65 years in Germany. J. Physiol. Anthropol..

[36] Queen R.M., Charnock B.L., Garrett W.E., Hardaker W.M., Sims E.L., Moorman C.T. (2008). A comparison of cleat types during two football-specific tasks on FieldTurf. Br. J. Sports Med..

[37] Imamura M., Imamura S.T., Salomão O., Pereira C.A.M., De Carvalho A.E., Neto R.B. (2002). Pedobarometric evaluation of the normal adult male foot. Foot Ankle Int..

